# Comparative analysis of physiological and biochemical traits in tobacco leaves of varying quality during development

**DOI:** 10.3389/fpls.2025.1653539

**Published:** 2025-11-18

**Authors:** Yue Yang, Ou Chen, Weimin Guo, Yunfei Ma, Yi Chen, Yonglei Jiang, Nan Shi, Dongfang Zheng, Wanpeng Xi, Binbin Hu

**Affiliations:** 1 Yunnan Academy of Tobacco Agricultural Sciences, Kunming, China; 2 College of Horticulture and Landscape Architecture, Southwest University, Chongqing, China; 3 Tobacco Agriculture Laboratory, Zhengzhou Tobacco Research Institute of China National Tobacco Corporation (CNTC), Zhengzhou, China; 4 Guizhou Academy of Tobacco Science, Guiyang, China; 5 Chuxiong State Tobacco Monopoly Bureau, Chuxiong, China

**Keywords:** developmental stage, low-quality tobacco, physiological traits, biochemical traits, key aroma components

## Abstract

**Introduction:**

During tobacco growth and development, variations in environmental conditions and cultivation practices can lead to the emergence of low-quality tobacco leaves (multi fertilizer and reviving tobacco leaves). Although most experiments have focused on the late roasting process of tobacco and related metabolites, few have reported the physiological and biochemical changes in the internal development stage of tobacco.

**Methods:**

Comparative analyses of physiological, colorimetric, pigment, and routine chemical parameters across normal mature tobacco leaves and low-quality tobacco leaves revealed significant disparities in growth vigor, chromatic evolution, pigment accumulation, and primary metabolite profiles. At the same time, the ultrastructural changes of leaf cells were observed by electron microscope to reveal the dysfunction at the cellular level.

**Results:**

This study investigated physiological and biochemical differences between normal mature tobacco leaves and low-quality tobacco leaves during their growth stages. Results showed that normal mature tobacco leaves exhibited significantly lower a* values but higher L* and b* values compared to low-quality tobacco leaves. Additionally, the contents of β-carotene, lutein, and chlorophylls a/b in three tobacco types decreased through developmental stages. Normal mature tobacco leaves exhibited lower plastid pigments than low-quality tobacco leaves at all stages. The contents of starch, reducing sugar, and total sugar in normal mature tobacco leaves reached the highest levels of 45.98%, 25%, and 27.65%, respectively, which are generally higher than those in low-quality tobacco leaves. At the same time, the contents of protein, total phytoalkaloid, and total nitrogen in most parts of normal mature tobacco leaves were the lowest, reaching as low as 2.7%, 0.07%, and 0.97%. Chlorine and potassium levels were more balanced in normal mature tobacco leaves than in low-quality ones.

**Discussion:**

Different quality tobacco leaves show different growth characteristics and chemical composition changes in the development process. Future research should investigate cultivation practices (e.g., fertilization timing, topping protocols) to optimize leaf phenotype and quality, thereby informing sustainable tobacco production strategies.

## 1 Introduction

Tobacco (*Nicotiana tabacum L.*) serves not only as a vital cash crop in China and numerous global economies (Yang et al., 2023), but also a key model plant for scientific research. During its development, intrinsic substance accumulation and quality formation critically determine the flavor and quality of processed tobacco products. Freshly harvested tobacco leaves frequently exhibit quality defects—including impurities, heightened irritancy, and off-flavors—that compromise usability (Wang et al., 2015). Notably, chemical composition in fresh leaves strongly correlates with post-curing quality metrics: appearance, aroma component integrity, and sensory evaluation outcomes (Zheng et al., 2019). Comparative analysis of physiological-biochemical traits across leaf quality types (normal mature, multi fertilizer and reviving tobacco leaves) during development provides critical insights into growth dynamics. These studies elucidate intrinsic regulatory patterns, directly supporting advancements in cultivation practices and genetic improvement strategies for premium tobacco production.

Multi fertilizer tobacco leaves (“black burst” leaves) develop deep green or yellowish-green color, rigid texture, and reduced elasticity near maturity. This occurs due to excess nitrogen fertilizer or poor environmental conditions (Zhu et al., 2011). These leaves exhibit chemical imbalances, typically marked by elevated nitrogenous compounds (e.g., proteins, alkaloids) and reduced soluble sugars, which persist post-curing and resulted in “greenish cured leaves”.

When mature tobacco plants take in too much nitrogen from their surroundings, their leaves start to turn greener, become thick and juicy, and lose the usual features of fully grown leaves. This process is called “reviving tobacco leaves.” This phenomenon manifests as excessive vegetative vigor, and resistance to natural chlorophyll degradation. Once leaves exhibit reviving characteristics, they are highly recalcitrant to re-entering normal maturation stages within a feasible time frame. Premature harvesting in these conditions leads to poor curing outcomes. These include chlorophyll-retaining veins and grayish-brown discoloration, which collectively compromise the sensory and commercial quality of cured tobacco. Multi fertilizer and reviving tobacco leaves are both types of low-quality tobacco. These phenomena are especially common in China’s tobacco farming, where farmers often overuse nitrogen fertilizers to boost harvests, even if it reduces quality (Huang et al., 2008). This agronomic imbalance exacerbates nicotine accumulation, disrupts carbon-nitrogen metabolism, and promotes the formation of both multi fertilizer and reviving tobacco leaves. As a result, the overall quality of tobacco leaves declines.

Topping is a pivotal cultural practice in tobacco field production, exerting a profound influence on plant architecture, nutrient partitioning, and leaf quality. By removing the apical meristem, topping abolishes apical dominance, redirecting dry matter accumulation toward leaf development and enhancing nutrient use efficiency (Jing, 2023). Topping serves as a cornerstone strategy for canopy management, nutritional regulation, and high-quality, yield-optimized tobacco production. As a deliberate growth perturbation, topping orchestrates hormonal and metabolic shifts. These enhance leaf biomass allocation and suppress vegetative overgrowth.

The chemical composition of tobacco leaves forms the basis of intrinsic quality. Key constituents (total sugars, reducing sugars, total nitrogen, total alkaloids, chlorine, potassium) are monitored as “conventional indices” for their quality impact (Tan and Qin, 2006). These components are intricately linked to sensory attributes (Chen et al., 2014; Cui, 2017; Xue et al., 2016) and combustion characteristics. For instance, plastid pigments (Yang et al., 2016) and free amino acids (Huang et al., 2017; Xiao et al., 2013; Yang and Luo, 2013) in fresh leaves correlate strongly with post-curing leaf appearance, smoking quality, and aroma precursor levels. Carbon metabolism during leaf maturation primarily focuses on total sugars, reducing sugars, and starch (Jia et al., 2022), which collectively constitute carbohydrate compounds. In contrast, nitrogen metabolism yields nitrogenous compounds (proteins, free amino acids, alkaloids), which profoundly impact sensory perception and consumer health risks. Mineral elements are indispensable for photosynthesis, nutrient translocation, and stress tolerance (Liu et al., 2010), regulating critical physiological processes. Therefore, comparative analysis of conventional chemical profiles among normal mature, multi fertilizer, and reviving tobacco leaves across developmental stages enables elucidation of metabolic disruptions impairing normal leaf maturation. Such insights are pivotal for flavor enhancement, quality improvement, and yield optimization in tobacco cultivation.

Currently, there’s still not enough global research on why low-quality tobacco leaves form during farming and how their physical and chemical processes change as they grow. This knowledge deficit risks compromising flue-cured tobacco quality and yield due to unmitigated metabolic disruptions in suboptimal leaves. To address this, the present study aims to compare physiological and biochemical traits among normal mature, multi fertilizer, and reviving tobacco leaves across key developmental stages. Characterize ultrastructural alterations in leaf cells via electron microscopy to elucidate cellular-level dysfunction. Analyze correlations between metabolic deviations and impaired leaf maturation, thereby providing scientific foundations for flavor enhancement and quality improvement in tobacco.

## 2 Materials and methods

### 2.1 Plant materials

This study was conducted in Chengjiang City, Yuxi Prefecture, Yunnan Province (23°19′—24°53′N, 101°16′—103°09′E) during the 2024 growing season. The experimental site features a subtropical plateau monsoon climate, characterized by an annual mean temperature range of 16.4 - 24.6°C. The region exhibits mild winters without severe cold and temperate summers without extreme heat, maintaining spring-like conditions year-round with distinct wet and dry seasons.

The trial utilized the locally cultivated tobacco variety Yunyan 300, with key phenological stages occurring on April 5 (transplanting) and July 9 (topping). Three experimental treatments were established based on regional fertilization practices:control group (standard fertilization protocol)、”Multi fertilizer” treatment and “Reviving” treatment. Detailed fertilization parameters for each treatment group are systematically presented in **Table 1**.

**Table 1:** Record of fertilization of Yunyan 300 in 2024.

Group name	Nitrogen consumption	Base fertilizer (12:6:24)	Seedling fertilizer (28:0:5)	Top application (12:6:24)	supplementary fertilizer (12:6:24)
Y300-CK	8.25 g/m^2^	18 g/m^2^	7.5 g/m^2^	36.3 g/m^2^	
Fertilization date		May 2	May 12	May 17	
Y300-Multi fertilizer	14.25 g/m^2^	18 g/m^2^	7.5 g/m^2^	61.2 g/m^2^	25.05 g/m^2^
Fertilization date		May 2	May 12	May 17	June 22
Y300-Reviving	14.25 g/m^2^	18 g/m^2^	7.5 g/m^2^	61.2 g/m^2^	25.05 g/m^2^
Fertilization date		May 2	May 12	May 17	July 10

The above ratios of compound fertilizer are all N: P2O5: K2O ratio.

### 2.2 Sampling and testing requirements

Samples were collected and tested at five time points at 7-day intervals, specifically: two weeks before topping (June 25), one week before topping (July 2), at the topping stage (July 9), one week after topping (July 17), and two weeks after topping (July 24). Each time, 30 disease-free tobacco leaves were selected and divided into three groups (A, B, C), with 10 leaves per group to serve as biological replicates. For each sampling instance, the selected location is the fifth leaf from the top of the topping site on individual tobacco plants. Each leaf was rapidly sectioned into five parts (Leaf tip, mid-leaf, lower leaf, midvein, and lateral veins), immediately frozen in liquid nitrogen and pooled. Subsequently, each group was further divided into two equal portions, which were labeled with the sampling time, tobacco leaf variety designation, and specific leaf section. Then stored at -80°C for subsequent analysis of conventional chemical constituents (including total sugars, reducing sugars, starch, total nitrogen, total alkaloids, protein, chlorine, and potassium) as well as chlorophyll (a and b), β-carotene, and lutein contents in different leaf sections. Additionally, for each treatment condition (normal, multi fertilizer, and reviving), three additional leaves were photographed on each sampling occasion to measure leaf length, width, and thickness. Thereafter, a portable colorimeter was employed to assess colorimetric values (L, a, b values) across various leaf sections, followed by histological examination of leaf cell structures post-sectioning.

### 2.3 Determination of pigment content

The determination of pigment content follows the method outlined in YC/T382-2010:Tobacco and tobacco products—Determination of plastid pigments—High performance liquid chromatography method (National tobacco standardization technical committee cigarette sub-technical committee, 2010). Freshly freeze-sampled tobacco leaves were freeze-dried at -45°C for 48 h using a lyophilizer. The dried material was subsequently pulverized and sieved through a 40-mesh sieve. Precisely 0.2 g of the resulting powder (weighed to the nearest 0.0001 g) was then transferred into a 50 mL Erlenmeyer flask. To this, 25 mL of extraction solvent (95% acetone solution) is added, followed by ultrasonic extraction for 20 minutes. After extraction, the solution is allowed to reach room temperature. An appropriate volume of the extracted solution is filtered through an organic membrane filter, and the filtrate is transferred into a 2 mL amber sample vial for high-performance liquid chromatography (HPLC) analysis. Chromatographic column: 150mm×3.9mm (inner diameter), stationary phase: C18, packed with 4 μm particles, mobile phase A: isopropanol, mobile phase B: 80% acetonitrile. Flow rate: 0.5 mL/min. Column temperature: 30°C. Injection volume: 10 μL. UV-Vis detector: detection wavelengths: chlorophyll a: 448 nm, other phytoplasmic pigments: 428 nm. The dry-basis chlorophyll-related pigment content is calculated using the formula:


X=25c/m(1−w)

X—Plastid pigment content (μg/g),

C—Pigment concentration in the extracted solution (μg/mL),

m—Mass of the sample(g),

w—Moisture content of the sample (%).

Results are reported as the mean of two parallel determinations, rounded to 0.01 μg/g. The relative deviation between parallel measurements must be less than 10%.

### 2.4 Determination of carbohydrate compound content

#### 2.4.1 Determination of total sugars and reducing sugars

Test samples are prepared according to YC/T31-1996 (National tobacco standardization technical committee, 1996). The tobacco leaves drying method required for the determination of various substances below is consistent with this. Randomly select a portion of tobacco leaves from each sample section and place them in an oven at no more than 40 °C until they can be crushed with fingers. Immediately grind the dried leaves after removal from the oven, with continuous grinding time not exceeding 2 minutes. Sieve the material; any unfiltered fine veins should be re-ground and sieved again. Transfer the sieved powder to a clean, dry wide-mouthed bottle and seal it tightly. Shake thoroughly to mix evenly. This constitutes the prepared sample. Open the numbered clean weighing dish and place it in the event a (100 ± 1)°C for 2 hours. Remove the dish, place it in a silica gel desiccator, and cool to room temperature (approximately 30 minutes). Weigh immediately as m0, accurate to 0.001g. Add 2-3g of the sample to the weighing dish and weigh as m1, accurate to 0.001g. Open the weighing dish and place it in the oven again. Arrange the dishes in layers using only the central shelf, spacing them 275cm apart. Dry at (100 ± 1)°C for 2 hours. Remove the dish, place it in a silica gel desiccator, cool to room temperature (approximately 30 minutes), and weigh as m2, accurate to 0.001g. Calculate the moisture content percentage of the sample using the following formula:


$$\text{W}=\frac{{\text{m}}_{1}-{\text{m}}_{2}}{{\text{m}}_{1}-{\text{m}}_{0}}\times 100\%$$

W—Mass percentage of moisture in the sample,%;

m_0_—Mass of the weighing vessel, g;

m_1_—Total mass of the weighing vessel and sample before drying, g;

m_2_—Total mass of the weighing vessel and sample after drying, g.

The determination of total sugars and reducing sugars follows the method outlined in “Tobacco and tobacco products─Determination of water soluble sugars─Continuous flow method YC/T159-2019.”(National tobacco standardization technical committee cigarette sub-technical committee, 2019). Specifically, 0.25 g of the sample is weighed into a 50 mL stoppered conical flask with precision to 0.0001 g. Subsequently, 25 mL of 5% acetic acid solution is added, and the flask is sealed and agitated on a shaker (at a speed exceeding 150 rpm) for 30 minutes to facilitate extraction. The extracted solution is filtered through rapid qualitative filter paper, with the initial 2–3 mL of filtrate discarded. The subsequent filtrate is collected for analytical purposes.

A series of standard working solutions and the processed filtrate from the sample are analyzed using the instrument. The continuous flow analyzer consists of the following parts: sampler, proportional pump, dialyzer, heating tank, spiral tube, colorimeter with 410nm filter, data processing device, and heat dissipation device. If the sample concentration exceeds the range of the standard working solutions, dilution is required prior to measurement. The water-soluble sugar content is calculated using the following formula:


$$a=\frac{n\times c\times V}{m\times (1-W)\times 1000}\times 100\%$$

a—Water-soluble total (or reducing) sugar content on a dry basis (expressed as glucose equivalent), in percentage (%),

n—Dilution factor,

C—Instrumentally determined value of water-soluble total (or reducing) sugar in the extraction solution, in milligrams per milliliter(mg/mL),

V—Volume of the extraction solution, in milliliters (mL),

m— Mass of the sample, in grams (mg);

W—Moisture content of the sample, expressed as a mass fraction.

#### 2.4.2 Determination of starch

The determination of starch content follows the method outlined in “Tobacco and tobacco products—Determination of starch—Continuous flow method:YC/T216-2013 (National tobacco standardization technical committee cigarette sub-technical committee, 2013)”. Samples were prepared according to YC/T31, and their moisture content was subsequently measured. A precisely weighed 0.25 g sample was placed into a 50 mL G3 sintered glass funnel. Subsequently, 25 mL of an 80% ethanol-saturated sodium chloride solution was added to the funnel, which was then immersed in a 400 mL beaker containing an appropriate amount of water. Ultrasonic extraction (350 W power) was performed at room temperature for 30 minutes. After extraction, the funnel was removed, and the extraction solution was discarded by opening the stopcock. The sample residue within the funnel was rinsed with 2 mL of the 80% ethanol-saturated sodium chloride solution, and the rinsate was discarded by applying pressure with a double-bulb aspirator before closing the stopcock. The funnel was returned to the 400 mL beaker, and 15 mL of a 40% perchloric acid solution was added to the sample residue. Ultrasonic extraction (350 W power) was conducted at room temperature for 10 minutes. Following this, 15 mL of water was added to the funnel, mixed thoroughly, and the starch extract was collected in a 50 mL Erlenmeyer flask by opening the stopcock. A 5 mL aliquot of the extract was accurately transferred to a 50 mL volumetric flask and diluted to the mark with water, then mixed thoroughly for analysis. The continuous flow analyzer consists of the following parts: sampler, proportional pump, spiral tube, colorimeter with 570 nm filter, and data processing device.

The starch content (a) expressed on a dry basis (in percentage) was calculated using the following formula:


$$a=\frac{6\times c\times V}{m\times (1-w)\times 1000}\times 100\%$$

C—Instrumental observation value of starch in the sample solution (mg/mL),

V—Volume to which the sample solution was diluted (mL),

m—Mass of the sample (mg),

ω— Mass fraction of moisture in the sample (%).

### 2.5 Determination of nitrogenous compound content

#### 2.5.1 Determination of total alkaloid content

The determination of total alkaloid content follows the method outlined in”Tobacco and tobacco products—Determination of total alkaloids—Continuous flow (potassium thiocyanate) method:YC/T468-2021 (National tobacco standardization technical committee cigarette sub-technical committee, 2021)”. Samples were prepared according to YC/T31, and their moisture content was subsequently measured. A precisely weighed 0.25 g sample (to the nearest 0.0001 g) was placed into a 50 mL conical flask with a stopper. Then, 25 mL of water (or 5% acetic acid solution) was added, the stopper was secured, and the flask was shaken on a shaker (at a speed exceeding 150 rpm) for 30 minutes to extract the alkaloids. The extraction solution was filtered using rapid qualitative filter paper, and the initial few milliliters (2 mL–3 mL) of the filtrate were discarded. Collect the subsequent filtrate for continuous flow analyzer. The continuous flow analyzer consists of the following parts: sampler, proportional pump, spiral tube, dialysis tank, colorimeter with 460nm filter, and data processing device.

The total alkaloid content (expressed as nicotine) on a dry basis (a), in percentage (%), was calculated using the following formula:


$$a=\frac{c\times v}{m\times (1-w)\times 1000}\times 100\%$$

C—Instrumental observation value of total alkaloids in the extraction solution (mg/mL),

v—Volume of the extraction solution (mL),

m—Mass of the sample (mg),

w—Mass fraction of moisture in the sample.

#### 2.5.2 Determination of protein content

The determination of protein content follows the method outlined in”Tobacco and tobacco products—Determination of protein—Continuous flow method:YC/T249-2008 (National tobacco standardization technical committee, 2008)”. Samples are prepared according to YC/T31, and moisture content is measured. Approximately 0.5 g of the sample is weighed into a 100 mL conical flask, with precision to 0.0001 g. Twenty-five milliliters of acetic acid solution is added, and the mixture is gently heated, maintaining boiling for 15 minutes. The solution is rapidly filtered through quantitative filter paper in a vacuum filtration apparatus, and the conical flask and precipitate are rinsed with acetic acid solution until the filtrate is colorless. The filter paper and precipitate are then transferred to a digestion tube. To the digestion tube, 0.1 g of mercury(II) oxide, 1.0 g of potassium sulfate, and 5 mL of concentrated sulfuric acid are added. The digestion tube is placed on a digestion apparatus for digestion. The following operating parameters for the digestion apparatus are recommended: maintain at 150 °C for 1 hour, then increase the temperature to 370 °C and maintain for 4 hours. After digestion, the tube is allowed to cool slightly, and a small amount of water is added. Once cooled to room temperature, the volume is adjusted to the mark with water, and the solution is thoroughly mixed. The continuous flow analyzer consists of the following parts: digester, recommended digestive tube capacity of 75mL, sampler, proportional pump, dialyzer, heating tank, spiral tube, colorimeter with 660nm filter, recorder or other suitable data processing device.

The protein content on a dry basis, expressed as a mass fraction (w), is presented as a percentage (%) and calculated using the following formula:


$$\omega =6.25\times \frac{c}{m\times (1-\omega_{1})}\times 100\%$$

c—Total nitrogen mass in the sample(mg),

m—Sample mass(mg),

w_1_—Mass fraction of moisture in the sample(%),

6.25—Conversion factor between protein and total nitrogen.

#### 2.5.3 Determination of total nitrogen content

The determination of total nitrogen content follows the method outlined in”Tobacco and tobacco products—Determination of total nitrogen—Continuous flow method:YC/T161-2002.”(National tobacco standardization technical committee, 2002), Test samples are prepared according to YC/T31, and moisture content is measured. Approximately 0.1 g of the test material is weighed into a digestion tube, with precision to 0.0001 g. To the digestion tube, 0.1 g of mercury(II) oxide, 1.0 g of potassium sulfate, and 5.0 mL of concentrated sulfuric acid are added. The digestion tube is placed on a digestion apparatus for digestion. The operating parameters for the digestion apparatus are as follows: 150°C for 1 hour, followed by 370°C for 1 hour. After digestion, the tube is allowed to cool slightly, and a small amount of water is added. Once cooled to room temperature, the volume is adjusted to the mark with water, and the solution is thoroughly mixed. The continuous flow analyzer consists of the following parts: digester (recommended digestive tube capacity of 75ml), sampler, proportional pump, dialyzer, heating tank, spiral tube, colorimeter with 660nm filter, recorder or other suitable data processing device.

The total nitrogen content on a dry basis is derived using the following formula:


$$\text{The}\;\text{total}\;\text{nitrogen}\;\text{content}\;(\%)=\frac{c}{m\times (1-W)}\times 100\%$$

c—Instrument-observed value of total nitrogen in the sample solution(mg),

m—Mass of the test material(mg),

W—Moisture content of the test sample.

### 2.6 Determination of mineral element content

#### 2.6.1 Determination of chlorine content

The determination of chlorine content follows the method outlined in”Tobacco and tobacco products—Determination of chloride—Continuous flow method:YC/T162-2011 (National tobacco standardization technical committee, 2011)”. Test samples are prepared according to YC/T31, and their moisture content is measured. Approximately 0.25 g of the test sample is weighed into a 50 mL stoppered conical flask, with precision to 0.1 mg. To this, 25 mL of water is added, the flask is stoppered, and the mixture is extracted for 30 minutes on a shaker at a speed greater than 150 revolutions per minute (rpm). The extraction solution is filtered using rapid qualitative filter paper, discarding the first 2 mL to 3 mL of filtrate, and the subsequent filtrate is collected for analysis. The continuous flow analyzer consists of the following parts: sampler, proportional pump, spiral tube, dialysis tank, colorimeter with 460 nm filter, and data processing unit.

The chlorine content, denoted as “a,” is calculated on a dry basis and expressed as a percentage (%) using the following formula:


$$a=\frac{c\times V}{m\times (1-\omega )}\times 100\%$$

c—Instrument-observed value of chlorine in the extraction solution (mg/mL),

V—Volume of the extraction solution(mL),

m— Mass of the test sample(mg),

w—Mass fraction of moisture in the test sample, expressed as a percentage (%).

#### 2.6.2 Determination of potassium content

The determination of potassium content follows the method outlined in”Tobacco and tobacco products—Determination of Potassium—Continuous flow method:YC/T217-2007 (National tobacco standardization technical committee, 2007)”. The moisture content of the test sample is measured according to YC/T31. Approximately 0.25 g of the test material is weighed into a 50 mL stoppered conical flask, with precision to 0.0001 g. To this, 25 mL of water is added to the 50 mL stoppered conical flask, which is then stoppered and extracted for 30 minutes on a shaker. The extraction solution is filtered using qualitative filter paper, discarding the initial few milliliters of filtrate, and the subsequent filtrate is collected for analysis(A 5% acetic acid solution may also be used as the extraction solution.). The continuous flow analyzer consists of the following parts: sampler, proportional pump, spiral tube, flame photometer detector, air compressor, liquefied gas, recorder or other data processing device.

The potassium content, denoted as “C,” is calculated on a dry basis and expressed as a percentage (%) using the following formula:


$$C=\frac{X\times V}{(m_{1}-m_{2})\times (1-W)\times 1000}\times 100\%$$

X— Instrument-observed value of potassium in the sample solution, in milligrams per milliliter (mg/mL),

V—Volume of the sample solution after dilution to a constant volume, in milliliters(mL),

W— Percentage moisture content (mass fraction) of the test sample, expressed as a percentage (%),

m_1_— Combined mass of the weighing bottle and the sample, in grams (mg),

m_2_—Mass of the weighing bottle, in grams (mg).

### 2.7 Statistical analysis

Three parallel experiments (n) were performed and the results are expressed as mean ± standard deviation (SD). The datasets of the compounds were processed using Microsoft Excel software. The GraphPad Prism 10 package was used for two-way analysis of variance (ANOVA) and chart drawing. According to the Duncan’s test, different letters in the figures indicate significant differences between means with statistical significance defined at P< 0.05. Error bars in the figures represent the standard deviation (SD) (±), calculated from three biological replicates (n = 3).

## 3 Results

### 3.1 Growth status of tobacco leaves with different quality attributes across developmental stages

**Figure 1:**
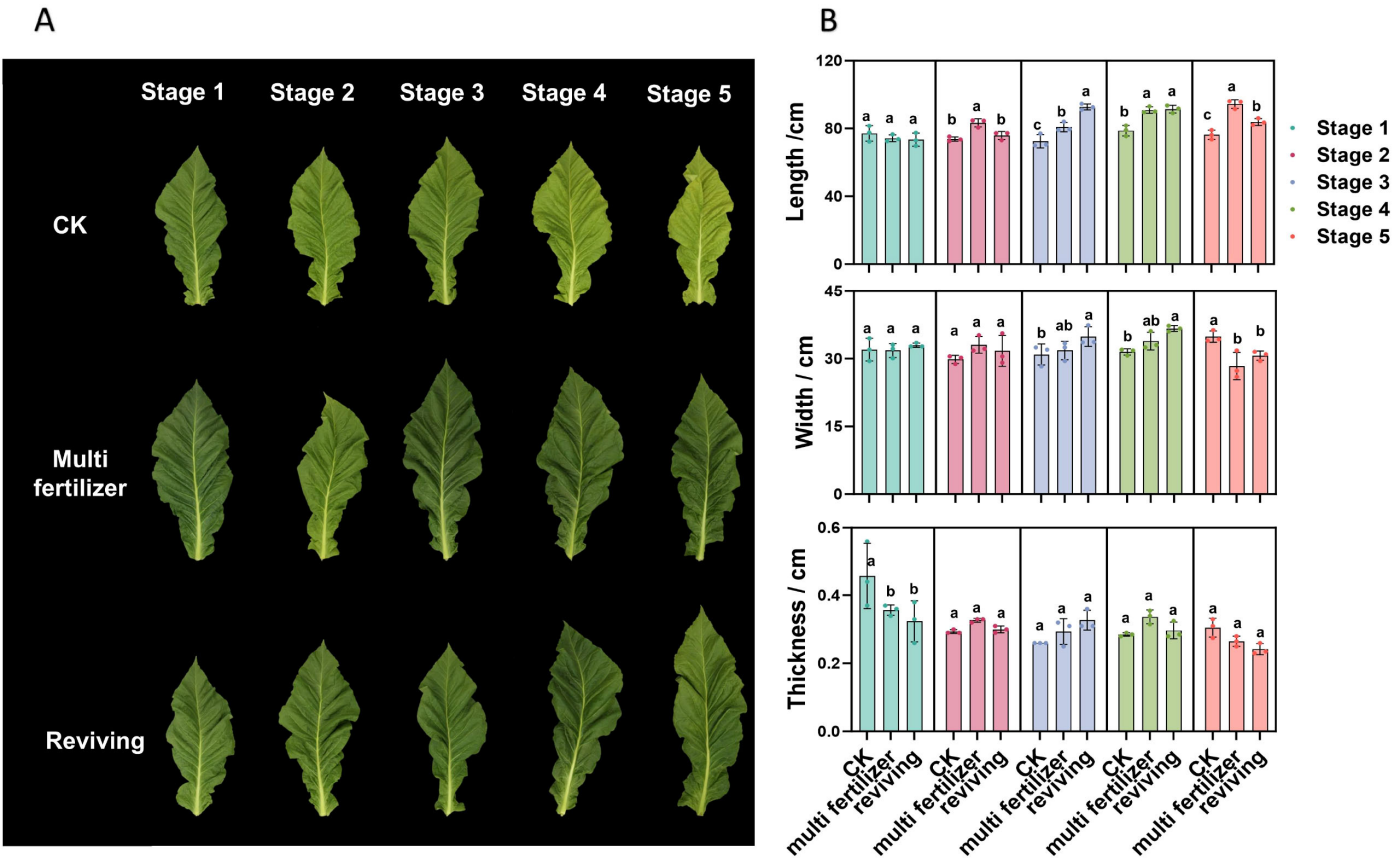
Growth status of tobacco leaves with different quality attributes across developmental stages. **(A)** Visual representation of the growth status of three tobacco leaf categories (normal, multi fertilizer, and reviving) across the five developmental stages. **(B)** Bar charts depicting the variations in leaf length, leaf width, and leaf thickness of the three tobacco leaf categories across the five developmental stages. a, b, and c represent the results of the significance difference analysis. If the groups do not contain the same letter, it indicates a significant difference between the groups, while if the groups contain the same letter, it indicates no significant difference between the groups.

During the developmental process, the growth conditions of various tobacco leaf types are depicted in **Figure 1A**. For normal tobacco leaves, the color gradually transitions from deep green to yellowish-green or yellow as they progress toward maturity. This color change is characterized by uniformity, accompanied by well-defined leaf veins and a relatively smooth leaf surface. In contrast, low-quality tobacco leaves—specifically, multi fertilizer and reviving tobacco leaves—display a notably deeper green hue compared to normal leaves. These low-quality leaves also exhibit more prominent leaf veins, reduced leaf surface evenness, and pronounced wrinkling.

**Figure 1B** illustrates the variations in leaf length, leaf width, and leaf thickness of the three different quality categories of tobacco leaves (normal, multi fertilizer, and reviving) across five developmental stages. Analysis of these data reveals that, during the developmental process, the leaf length and leaf width of all three quality categories generally exhibit an upward trend. Conversely, leaf thickness initially decreases from developmental stage 1 to stage 3, followed by a pattern of initial increase and subsequent decline from stage 4 to stage 5.

A further comparative examination of the three quality categories indicates that, during developmental stages 2 to 4, low-quality tobacco leaves (multi fertilizer and reviving) demonstrate greater leaf length, leaf width, and leaf thickness compared to normal mature tobacco leaves. However, at developmental stage 5, occurring two weeks after topping, both leaf width and thickness of low-quality tobacco leaves are reduced compared to normal mature tobacco leaves, while leaf length remains greater.

The developmental period is divided into five distinct stages: stage 1 (two weeks before topping), stage 2 (one week before topping), stage 3 (topping period), stage 4 (one week after topping), and stage 5 (two weeks after topping). Comparative analyses of leaf quality attributes across these stages were conducted using two-way analysis of variance (P< 0.05). Error bars represent standard deviation (n=3).

### 3.2 Color difference dynamics across leaf segments and quality attributes during tobacco development

**Figure 2:**
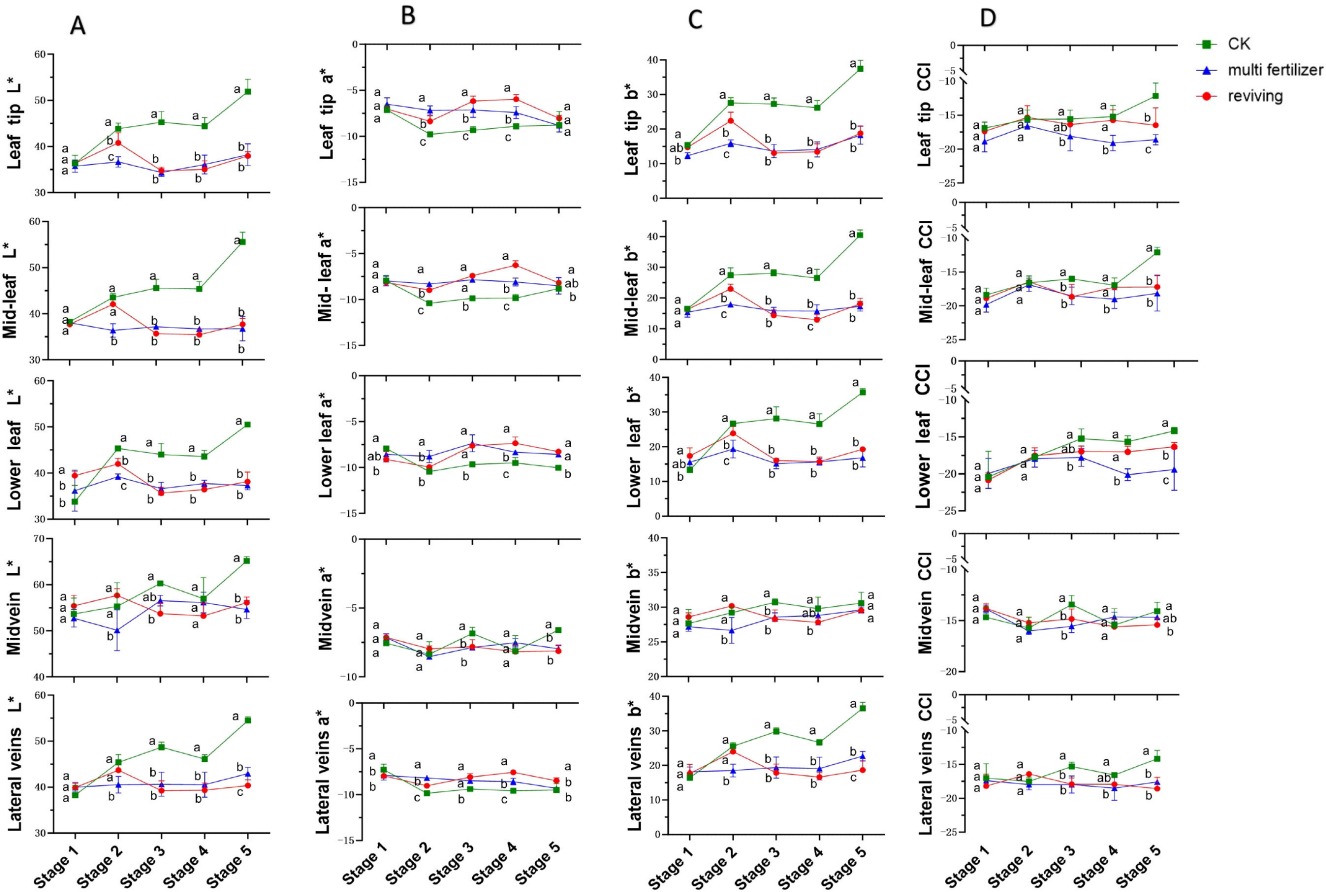
Line graphs depicting the temporal variations in L*(lightness), a*(red-green chromaticity), b*(yellow-blue chromaticity), and Color Consistency Index **(CCI)** values across five anatomical segments (e.g.,leaf tip, mid-leaf, lower leaf, midvein, lateral vein) of three tobacco leaf quality categories during the five developmental stages. **(A)** Temporal dynamics of L*(lightness) values. **(B)** Temporal dynamics of a*(red-green chromaticity) values. **(C)** Temporal dynamics of b*(yellow-blue chromaticity) values. **(D)** Temporal dynamics of Color Consistency Index (CCI) values. Comparative analyses of leaf quality attributes across these stages were conducted using two-way analysis of variance (P< 0.05). Error bars represent standard deviation (n=3). a, b, and c represent the results of the significance difference analysis. If the groups do not contain the same letter, it indicates a significant difference between the groups, while if the groups contain the same letter, it indicates no significant difference between the groups.

**Figure 2** illustrates the temporal variations in L* (lightness) values for five anatomical segments of three tobacco leaf quality categories (normal mature, multi fertilizer, and reviving) across developmental stages. For normal mature tobacco and reviving tobacco leaves, L* values in all segments generally followed an initial increase followed by a decline. However, normal mature tobacco leaves exhibited a less pronounced decline and a delayed onset of reduction compared to reviving tobacco leaves. In contrast, multi fertilizer tobacco leaves displayed a unique L* pattern in the main vein: an initial decrease, followed by an increase, and subsequent decline, while other segments showed relatively stable L* trends.

Longitudinal comparisons of L* values across stages 2–5 revealed that normal mature tobacco leaves maintained significantly higher L* values in all segments compared to multi fertilizer and reviving tobacco leaves. The L* differences between multi fertilizer and reviving tobacco leaves were largely non-significant, suggesting that normal mature tobacco leaves retained greater lightness during development. This aligns with their concurrent reduction in chlorophyll content, which typically correlates with brighter leaf appearance.

The a* parameter, representing red-green chromaticity, exhibited distinct trends across leaf quality categories. normal mature tobacco leaves demonstrated an overall a* decline followed by an increase during development. Multi fertilizer tobacco leaves showed relatively stable a* values, though most segments trended downward. Reviving tobacco leaves, however, followed a a* pattern of decline-increase-decline. Cross-sectional comparisons at developmental stages 2–4 revealed significantly lower a* values in normal mature tobacco leaves compared to multi fertilizer and reviving tobacco leaves across leaf tip, mid-leaf, lower leaf, and lateral vein segments. By stage 5, a* disparities diminished, indicating that normal mature tobacco leaves retained greener coloration during earlier developmental stages, consistent with enhanced chlorophyll accumulation.

The b* parameter, reflecting yellow-blue chromaticity, followed a general increase-decline-increase trajectory across all leaf quality categories, though low-quality leaves (multi fertilizer and reviving) exhibited a more pronounced b* decline. During early growth (low b* values), leaves displayed deep green coloration due to high chlorophyll concentrations. As leaves matured, chlorophyll degradation and carotenoid (e.g., lutein, β-carotene) accumulation elevated b* values, shifting leaf color toward yellow. Notably, normal mature tobacco leaves consistently registered significantly higher b* values than multi fertilizer and reviving tobacco leaves across most stages and segments, reaching a peak of 40.46 in mid-leaf segments at stage 5. This underscores that normal mature tobacco leaves undergo more pronounced yellowing during development.

The Color Consistency Index (CCI), a quantitative metric assessing color stability under standardized lighting protocols, was analyzed for all leaf segments. CCI values for multi fertilizer and reviving tobacco leaves were consistently lower than those of normal mature tobacco leaves across most developmental stages. This finding corroborates visual observations of reduced color variability in low-quality leaves, indicating less dynamic color transitions during maturation.

### 3.3 Pigment content dynamics during tobacco leaf development

**Figure 3:**
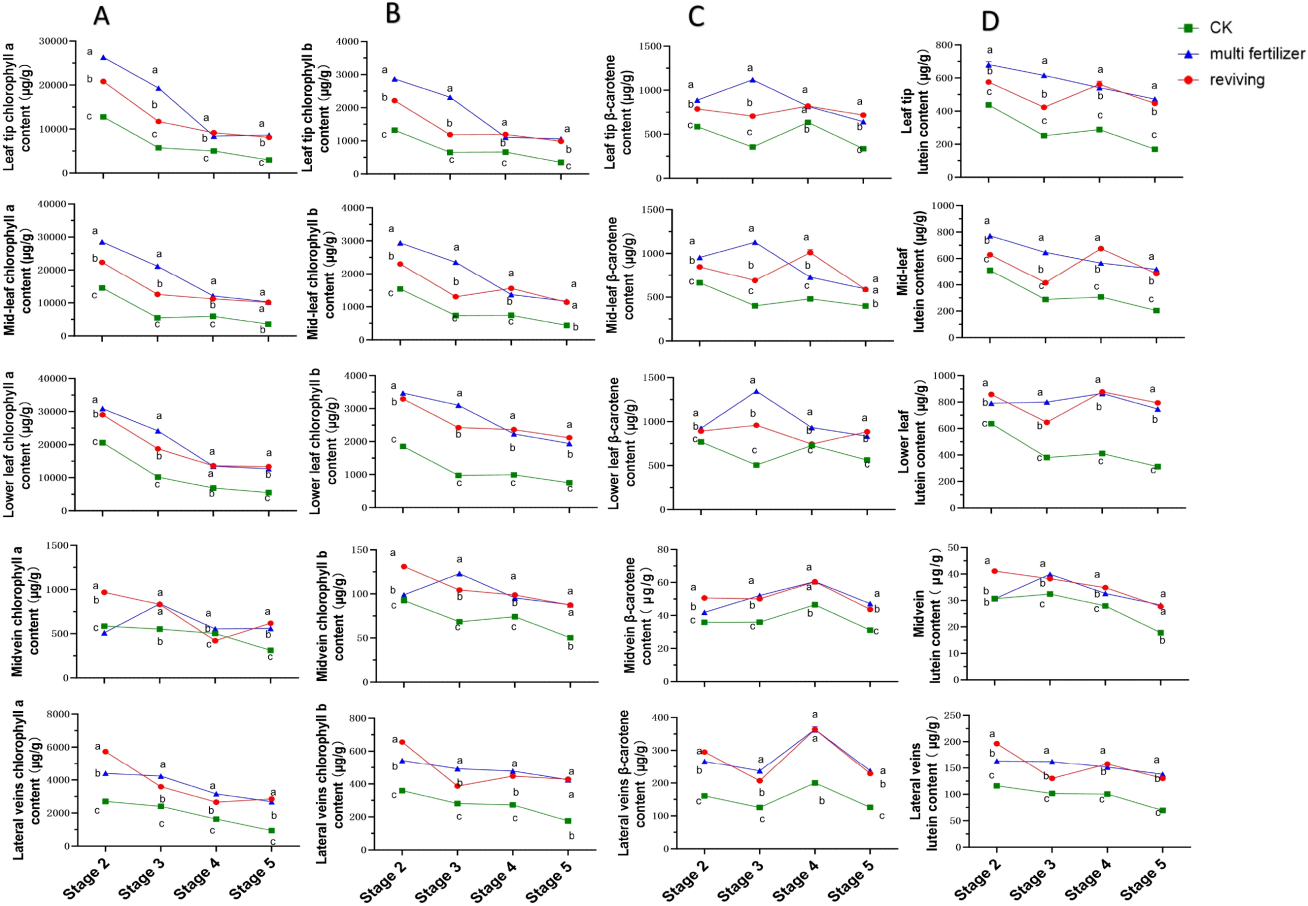
Line graphs illustrating the temporal dynamics of chlorophyll a (Chl a), chlorophyll b (Chl b), β-carotene, and lutein contents across five anatomical segments of three tobacco leaf quality categories during the five developmental stages. **(A)** Temporal dynamics of chlorophyll a (Chl a) content. **(B)** Temporal dynamics of chlorophyll b (Chl b) content. **(C)** Temporal dynamics of β-carotene content. **(D)** Temporal dynamics of lutein content. Comparative analyses of leaf quality attributes across these stages were conducted using two-way analysis of variance (P< 0.05). Error bars represent standard deviation (n=3). a, b, and c represent the results of the significance difference analysis. If the groups do not contain the same letter, it indicates a significant difference between the groups, while if the groups contain the same letter, it indicates no significant difference between the groups.

The temporal trends in chlorophyll a (Chl a) content across five anatomical segments of three tobacco leaf quality categories (normal mature, multi fertilizer, and reviving) exhibited similar declining patterns during development, with the most pronounced reductions occurring between stages 2-4 (**Figure 3A**). In early developmental stages (high Chl a levels), leaves displayed deep green coloration. As maturation progressed, Chl a content gradually diminished, reaching a minimum of 311.43 µg/g in the midvein of normal mature tobacco leaves at stage 5. Further analysis revealed that normal mature tobacco leaves consistently maintained lower Chl a levels across all segments and stages compared to the two low-quality categories, except in the midvein. Notably, differences in Chl a content between multi fertilizer and reviving tobacco leaves diminished during stages 4-5, suggesting that low-quality leaves accumulate greater Chl a during development.

The dynamics of chlorophyll b (Chl b) content mirrored those of Chl a, showing a general decline across all leaf segments and quality categories(**Figure 3B**). The steepest Chl b reduction occurred between stages 23, with the lowest value (50.28 µg/g) recorded in the midvein of normal mature tobacco leaves at stage 5. Comparative analysis confirmed that normal mature tobacco leaves exhibited lower Chl b levels in all segments and stages compared to multi fertilizer and reviving tobacco leaves, reinforcing the observation that low-quality leaves retain higher Chl b accumulation during development. This finding aligns with the Chl a results, indicating a consistent pigment storage pattern in low-quality leaves.

β-Carotene trends diverged among leaf quality categories. normal mature tobacco and reviving tobacco leaves followed a decline-increase-decline pattern across segments, while multi fertilizer tobacco leaves displayed an initial increase followed by a decline(**Figure 3C**). This suggests a progressive reduction in β-carotene during late developmental stages. Longitudinal comparisons revealed that normal mature tobacco leaves consistently registered lower β-carotene levels than low-quality leaves across all segments and stages, supporting the hypothesis that low-quality leaves accumulate greater β-carotene during maturation.

Lutein content trends varied by leaf quality category (**Figure 3D**). normal mature tobacco leaves exhibited an overall decline, with slight increases in apical, mid, and basal segments during stage 3 (though these were minor). Multi fertilizer tobacco leaves displayed a general decline in apical, mid, and lateral vein segments but an initial increase followed by a decline in basal and main vein segments. Reviving tobacco leaves followed a decline-increase-decline pattern across most segments. Notably, normal mature tobacco leaves maintained lower lutein levels than low-quality leaves in all segments and stages, indicating that low-quality leaves synthesize and retain higher lutein concentrations during development. This outcome is consistent with the trends observed for Chl a, Chl b, and β-carotene, reinforcing a broader pattern of enhanced pigment accumulation in low-quality leaves.

### 3.4 Changes in routine chemical components of tobacco leaves with different quality traits during development

#### 3.4.1 Starch and sugar content dynamics

**Figure 4:**
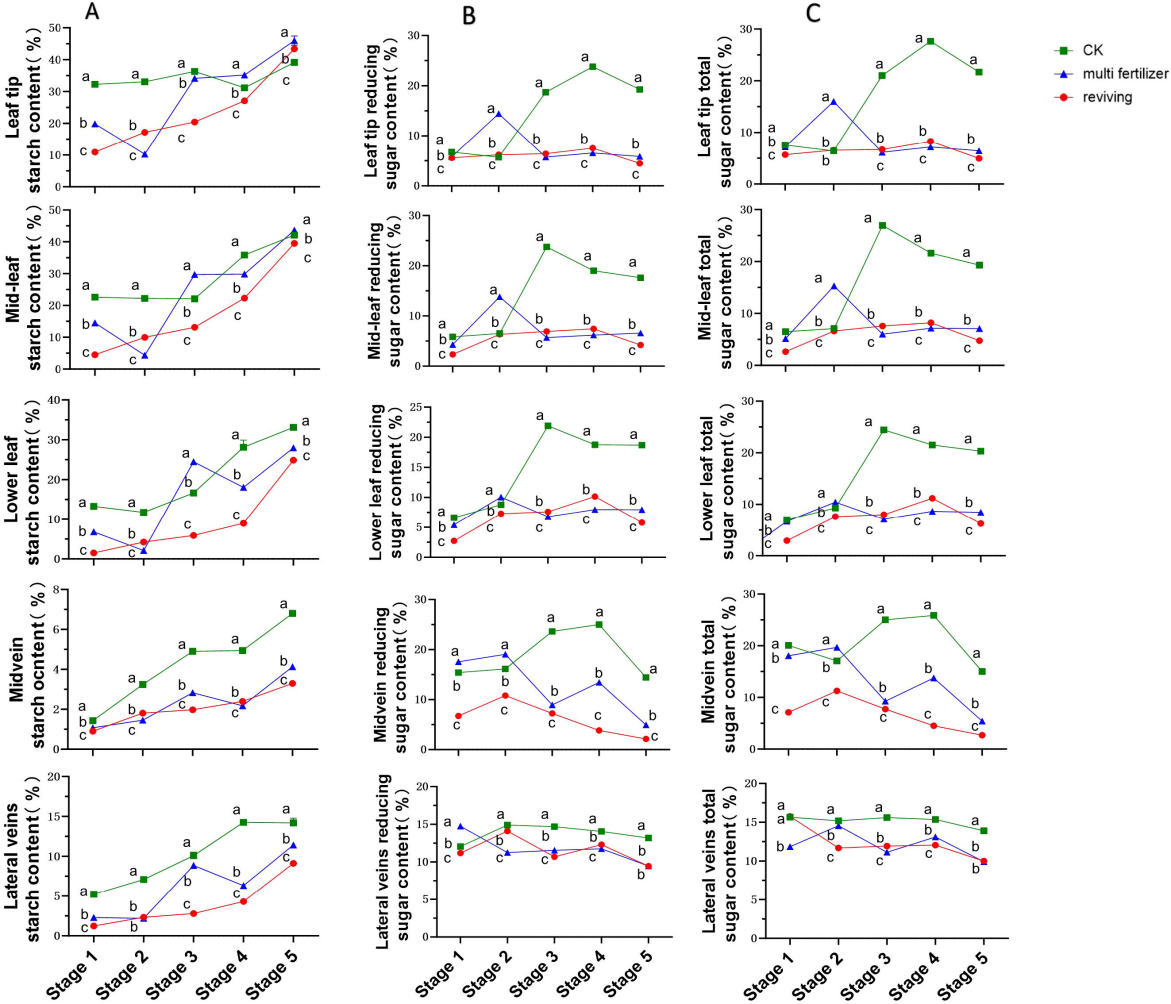
Line graphs depicting the temporal dynamics of starch, reducing sugar, and total sugar contents across five anatomical segments of three tobacco leaf quality categories during five developmental stages. **(A)** Temporal dynamics of starch content. **(B)** Temporal dynamics of reducing sugar content. **(C)** Temporal dynamics of total sugar content. Comparative analyses of leaf quality attributes across these stages were conducted using two-way analysis of variance (P< 0.05). Error bars represent standard deviation (n=3). a, b, and c represent the results of the significance difference analysis. If the groups do not contain the same letter, it indicates a significant difference between the groups, while if the groups contain the same letter, it indicates no significant difference between the groups.

The temporal starch content patterns across anatomical segments of the three tobacco leaf quality categories exhibited broadly similar increasing trends, with notable exceptions in multi fertilizer tobacco leaves(**Figure 4A**). Specifically, starch levels in multi fertilizer tobacco leaves declined sharply during stages 2 and 4 across all segments. The lowest starch content (0.9%) was observed in the main vein of low-quality leaves (both multi fertilizer and reviving) at stage 1, while the highest value (45.98%) was recorded in leaf tip segment of multi fertilizer tobacco leaves at stage 5. Notably, normal mature tobacco leaves maintained higher starch concentrations than low-quality counterparts in most segments and stages, following the hierarchy: normal mature > multi fertilizer > reviving. This finding aligns with electron microscopy observations during post-harvest curing, confirming that normal mature tobacco leaves accumulate greater starch reserves during development.

Reducing sugar levels in normal mature tobacco leaves followed a rise-and-decline pattern across all segments, though trends in lateral veins were relatively stable (**Figure 4B**). Peak reducing sugar content in normal mature tobacco leaves reached approximately 25% in the main vein, significantly exceeding levels in low-quality leaves during the same stages. Both multi fertilizer and reviving tobacco leaves also exhibited initial increases followed by decreases, but these trends were less pronounced, particularly after stage 3, where fluctuations in multi fertilizer and reviving tobacco leaves diminished. From stage 2 onward, normal mature tobacco leaves consistently registered higher reducing sugar concentrations than low-quality leaves, with a delayed inflection point for reduction, suggesting superior reducing sugar accumulation during development.

Excluding lateral veins, total sugar dynamics in normal mature tobacco leaves mirrored those of reducing sugars, peaking and then declining, with apical segments reaching approximately 27%—far exceeding levels in low-quality leaves during the same stages(**Figure 4C**). Lateral veins in normal mature tobacco leaves showed minimal total sugar variation. Multi fertilizer tobacco leaves displayed a similar but less pronounced rise-and-decline pattern, while reviving tobacco leaves exhibited relatively stable total sugar levels with negligible fluctuations. Consistent with reducing sugar results, normal mature tobacco leaves maintained significantly higher total sugar concentrations than low-quality counterparts after stage 2, with a delayed inflection point for decline, confirming their superior capacity for total sugar accumulation during development.

#### 3.4.2 Changes in nitrogenous compound content during tobacco leaf development

Protein content trends across segments of the three leaf quality categories were broadly similar, showing declining patterns throughout development. normal mature tobacco leaves consistently exhibited lower protein concentrations than the two low-quality groups across all segments and stages. Within the low-quality categories, multi fertilizer tobacco leaves accumulated less protein than reviving tobacco leaves at all time points. The protein accumulation hierarchy was thus: reviving > multi fertilizer > normal mature, confirming that low-quality leaves (especially reviving) retain higher protein reserves during development.

**Figure 5:**
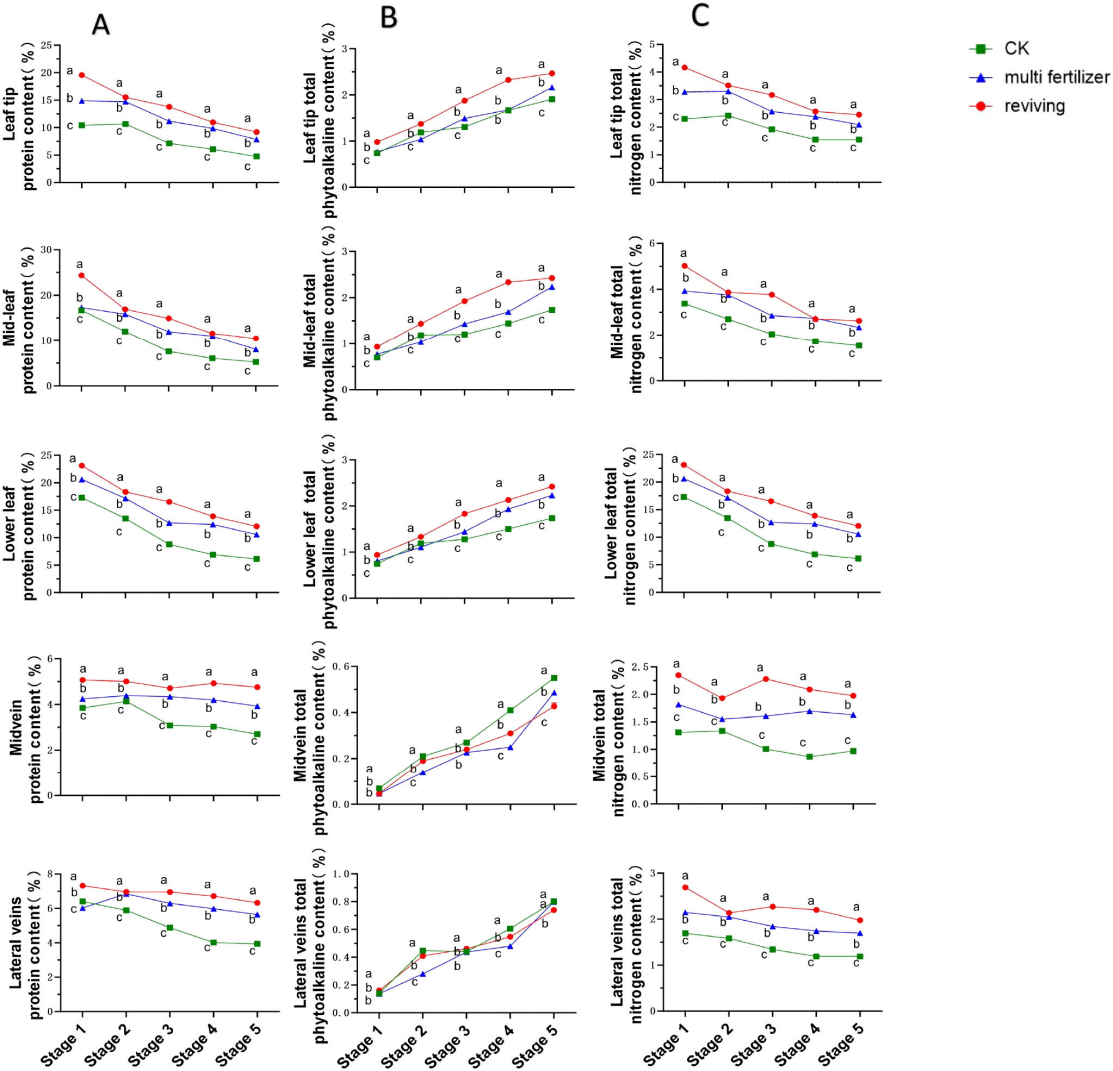
Line graphs depicting the temporal dynamics of protein, total alkaloid, and total nitrogen contents across five anatomical segments of three tobacco leaf quality categories during the five developmental stages. **(A)** Temporal dynamics of protein content. **(B)** Temporal dynamics of total phytoalkaloid content. **(C)** Temporal dynamics of total nitrogen content. Comparative analyses of leaf quality attributes across these stages were conducted using two-way analysis of variance (P< 0.05). Error bars represent standard deviation (n=3). a, b, and c represent the results of the significance difference analysis. If the groups do not contain the same letter, it indicates a significant difference between the groups, while if the groups contain the same letter, it indicates no significant difference between the groups.

The temporal trends in total phytoalkaloid content across anatomical segments (leaf tip, mid-leaf, lower leaf, midvein, lateral vein) of the three tobacco leaf quality categories exhibited broadly similar increasing patterns (**Figure 5B**). During most developmental stages, normal mature tobacco leaves registered lower total phytoalkaloid concentrations in the leaf tip, mid-leaf, lower leaf segments compared to the two low-quality groups. Among the low-quality categories, multi fertilizer tobacco leaves accumulated less total phytoalkaloid than reviving tobacco leaves in all segments. However, in the midvein and lateral veins, normal mature tobacco leaves displayed the highest total phytoalkaloid levels, followed by reviving and then multi fertilizer tobacco leaves, which had the lowest concentrations in these vascular regions. These findings suggest that low-quality leaves accumulate greater total phytoalkaloid in the leaf tip, mid-leaf, lower leaf segments during development.

Total nitrogen content trends closely mirrored those of protein content, with declining patterns observed across all segments and stages. normal mature tobacco leaves maintained significantly lower total nitrogen levels than low-quality leaves in all segments and developmental stages. The total nitrogen accumulation hierarchy followed the same order as protein: reviving > multi fertilizer > normal mature, providing evidence that low-quality leaves (particularly reviving) accumulate greater nitrogen during development.

#### 3.4.3 Changes in mineral element content during tobacco leaf development

**Figure 6:**
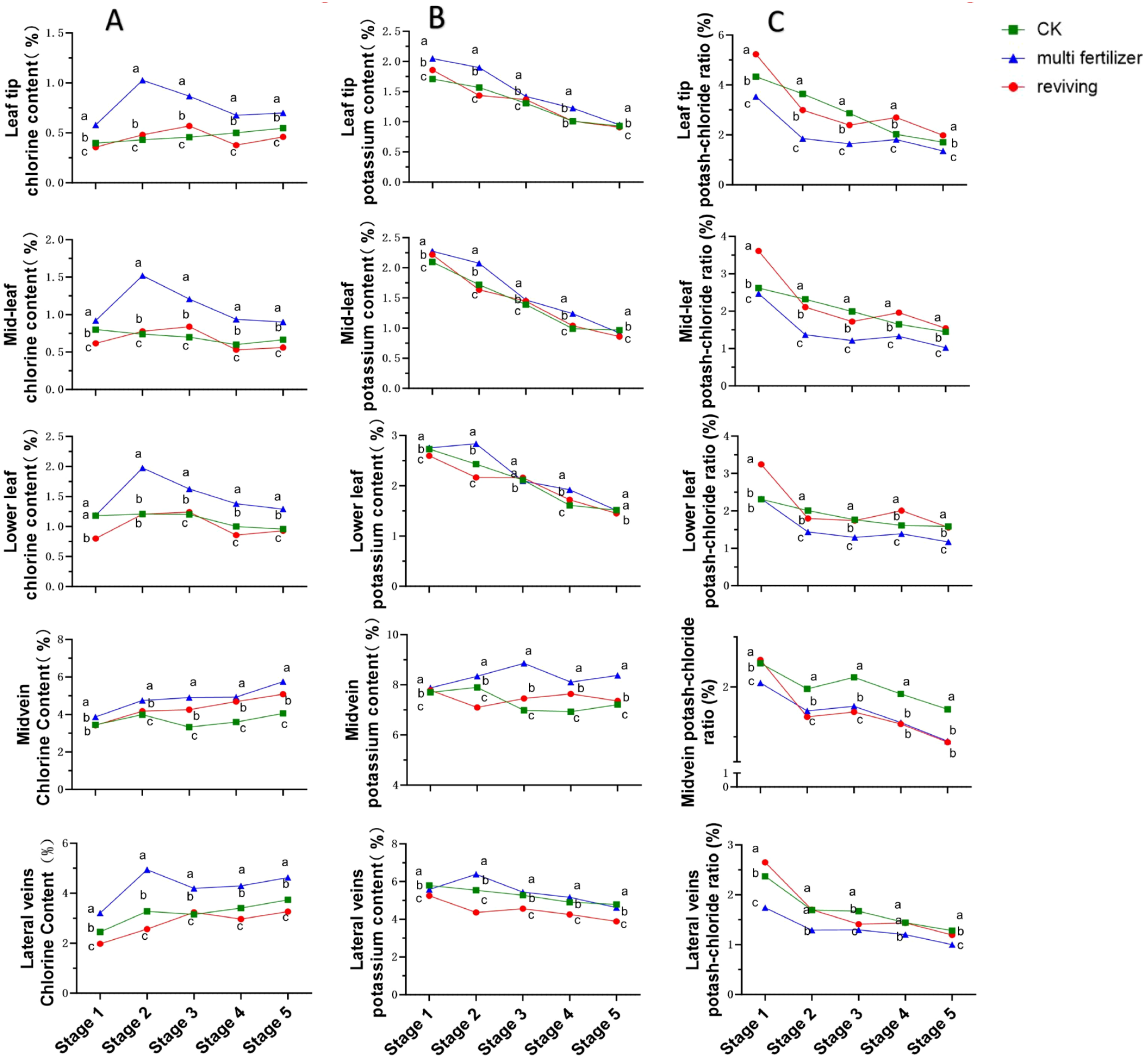
Line graphs illustrating the temporal dynamics of chlorine (Cl) content, potassium (K) content, and potassium-chlorine ratio across five anatomical segments of three tobacco leaf quality categories during the five developmental stages. **(A)** Temporal dynamics of chlorine (Cl) content. **(B)** Temporal evolution of potassium (K) content. **(C)** Temporal dynamics of potassium-chlorine ratio. Comparative analyses of leaf quality attributes across these stages were conducted using two-way analysis of variance (P< 0.05). Error bars represent standard deviation (n=3). a, b, and c represent the results of the significance difference analysis. If the groups do not contain the same letter, it indicates a significant difference between the groups, while if the groups contain the same letter, it indicates no significant difference between the groups.

The temporal trends in chlorine content across anatomical segments of the three tobacco leaf quality categories exhibited broadly similar patterns, characterized by an initial increase, followed by a decline, and a subsequent rise in most segments (**Figure 6A**). In all segments except the midvein, normal mature tobacco leaves maintained chlorine concentrations intermediate to the two low-quality groups across most developmental stages. multi fertilizer tobacco leaves consistently registered the highest chlorine levels in all segments at every developmental stage, indicating greater chlorine accumulation in these leaves during growth. In contrast, reviving tobacco leaves displayed the lowest chlorine content, while normal mature tobacco leaves exhibited intermediate chlorine accumulation.

Potassium content trends across the five segments also showed broad similarities, with a general declining pattern observed in the other segments excluding the main vein(**Figure 6B**). The lowest potassium concentration (0.86%) was recorded in the mid-leaf of reviving tobacco leaves during stage 5, while the highest concentration (8.86%) was detected in the midvein of multi fertilizer tobacco leaves during stage 3. Similar to chlorine, potassium levels in normal mature tobacco leaves remained intermediate between the two low-quality groups across most segments and stages, following the hierarchy: multi fertilizer > normal mature > reviving. These findings suggest that normal mature tobacco leaves maintain a moderate potassium accumulation profile during development, sandwiched between the hyper-accumulating multi fertilizer tobacco leaves and the hypo-accumulating reviving tobacco leaves.

As shown in **Figure 6C**, we can find that the potassium-chlorine ratio showed a downward trend during the growth and development of tobacco, and the potassium-chlorine ratio was usually the highest in normal mature tobacco leaves, while the potassium-chlorine ratio was the lowest in multi-fertilizer tobacco leaves, reaching the lowest of 1.00% in the lateral vein at stage 5.

### 3.5 Electron microscopic structural dynamics of tobacco leaves with varying quality during development

**Figure 7:**
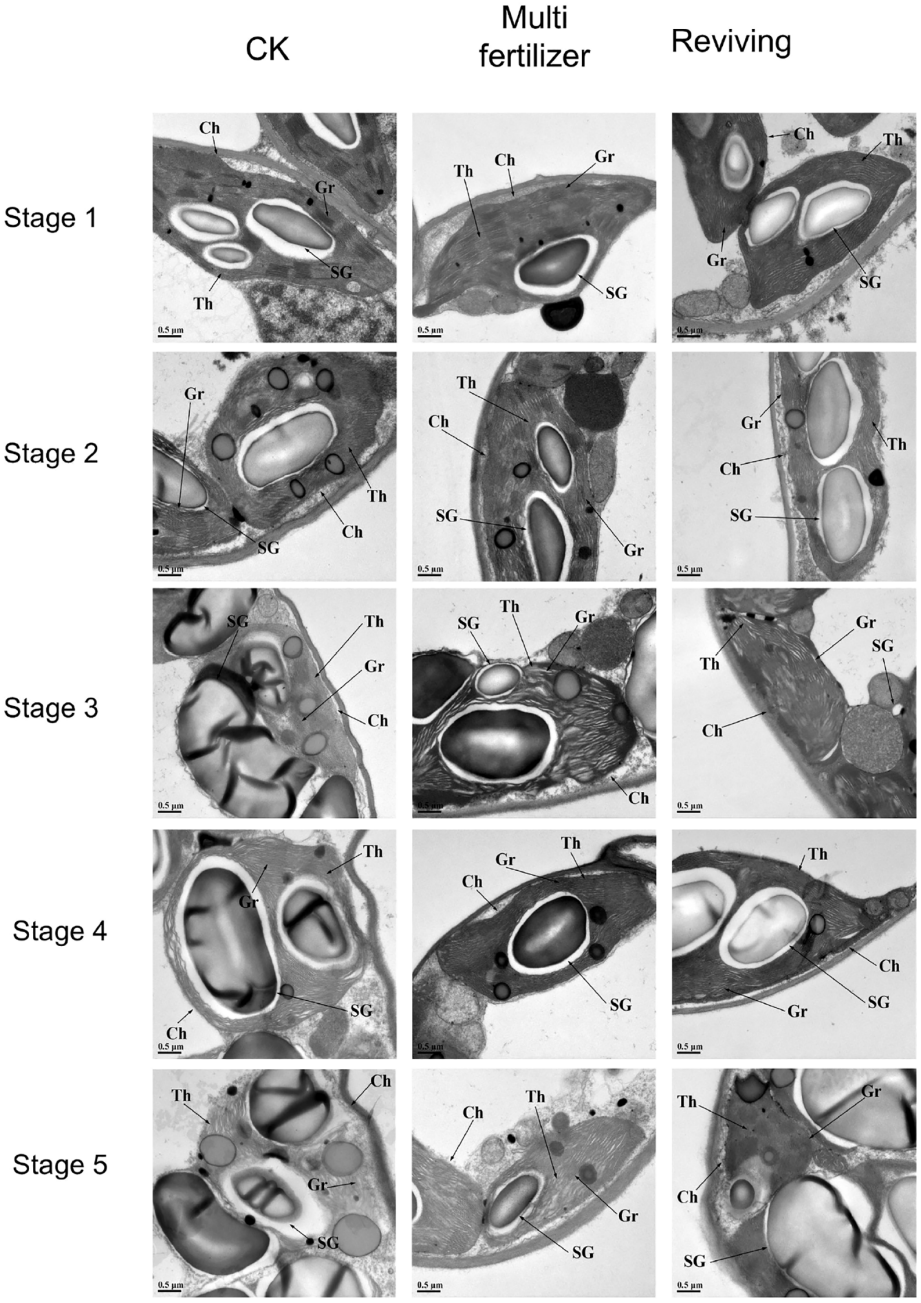
Electron microscopic (EM) depictions of chloroplast ultrastructure in three tobacco leaf quality categories across five distinct developmental stages (Zheng et al., 2020). SG, Starch granules; Th, Stroma thylakoids, Gr, Grana thylakoids; Ch, Chloroplast.

Electron microscopic (EM) observations of cellular structural alterations in tobacco leaves of three quality categories (normal mature, multi fertilizer, and reviving) across distinct developmental stages are illustrated in **Figure 7**. During the entire developmental trajectory, starch granule volume in normal mature tobacco leaves exhibited a trend of initial expansion followed by reduction, with partial granule dissociation observed. Under identical field-of-view conditions, normal mature tobacco leaves contained a higher abundance of starch granules compared to the two low-quality groups. Their thylakoid lamellae began disintegrating from stage 3, marked by reduced structural clarity and progressive lamellar loosening.

Throughout development, multi fertilizer tobacco leaves displayed significantly lower starch granule abundance and size relative to both normal mature and reviving tobacco leaves. Their thylakoid architecture was comparatively looser than normal mature tobacco leaves during early stages (stage 1), correlating with delayed leaf maturation. However, they retained relatively distinct chloroplast structures in later stages (stages 3-5).

1The thylakoid lamellae of the reviving tobacco leaves became more loose in stage 2 than in stage 1, and then gradually became clear in stage 3 and 4, and were relatively more regular and compact than those of normal mature tobacco leaves and multi fertilizer tobacco leaves. Starch granule dynamics in reviving tobacco leaves largely mirrored those of normal mature tobacco leaves, though their thylakoid lamellae began re-disintegrating in stage 5, with disassembly severity intermediate between normal mature and multi fertilizer tobacco leaves.

## 4 Discussion

Tobacco, a specialized psychoactive cash crop, exhibits critical quality and economic determinants governed by its internal biochemical composition and yield potential. The developmental stage surrounding topping (removal of the apical meristem) serves as a pivotal window for internal quality formation. During this time, normal mature tobacco leaves diverge significantly in physiological and biochemical traits from two low-quality phenotypes: multi fertilizer and reviving tobacco leaves.

### 4.1 Color difference and pigment content dynamics

Leaf color changes serve as a critical visual indicator of tobacco maturity status (Jia et al., 2022). Visual inspection of cured leaf can provide suitable insight for leaf chemistry (nicotine and reducing sugar concentration) and usability in cigarettes (Sun et al., 2011). Zhang et al. (2013) and Huo et al. (2011) employed colorimetric analysis to investigate pigment content fluctuations in conventional tobacco cultivars during fresh leaf maturation and curing, revealing strong correlations between pigment levels and colorimetric values. Leaf coloration fundamentally reflects shifts in plastid pigment composition (Jia et al., 2022), with plastid pigments (primarily chlorophylls and carotenoids) playing critical roles in determining leaf quality and usability (Shao et al., 2007). The abundance and ratios of these pigments profoundly influence both visual aesthetics (appearance) and intrinsic attributes (flavor, combustibility) of tobacco leaves. Chlorophyll degradation, manifesting as leaf chlorosis, is a hallmark of senescence, linked to thylakoid membrane disintegration in chloroplasts (Branka et al., 2016). Excessive nitrogen metabolism accelerates the expression of chloroplast degradation-related genes, disrupting normal senescence signaling pathways. Over-supply of nitrogen causes the expansion of chloroplast lamellar structures in mesophyll cells, exhibiting a ‘juicy’ characteristic. This structural abnormality directly weakens the photosynthetic capacity of chloroplasts. Our study Confirmed correlative trends between pigment content shifts and colorimetric (L*, a*, b*) dynamics across developmental stages. Luminance (L*) analysis revealed that normal mature leaves maintained elevated brightness throughout development, consistent with their lower chlorophyll content. Reviving tobacco leaves exhibited the steepest decline in a* during late developmental stages (stage 4-5), likely attributable to accelerated chlorophyll degradation and enhanced carotenoid accumulation. Additionally, normal mature tobacco leaves displayed consistently superior b* across most developmental stages and leaf zones, indicating greater yellowing intensity during senescence.

Chlorophyll a and b contents in normal mature tobacco leaves were consistently lower across all developmental stages and leaf zones compared to lower-quality phenotypes. This disparity may stem from differences in nitrogen uptake efficiency during late developmental stages, which influence physiological metabolism and photosynthetic performance. Normal mature tobacco leaves exhibited elevated β-carotene levels, suggesting enhanced antioxidant capacity and delayed senescence. Conversely, their reduced lutein content may significantly impact leaf yellowness and overall quality. These variations likely arise from differential regulation of pigment synthesis/degradation pathways, photosynthetic efficiency, and metabolic activity. Future studies should elucidate mechanistic links between these processes and tobacco quality to inform production practices and quality enhancement strategies.

### 4.2 Carbon-nitrogen metabolism dynamics

Carbon-nitrogen (C-N) metabolism represents the foundational physiological process governing tobacco leaf maturation (Zhang et al., 2014). Within the carbon metabolic pathway, starch and reducing sugars are key components. Chen et al. (2020) reported that, as tobacco leaves yellow during senescence, starch, total sugar, and reducing sugar contents exhibit a rise-and-fall trend. Similarly, Zhang et al. (2010) observed that during leaf maturation, photosynthetic efficiency declines, leading to weakened carbohydrate synthesis and enhanced degradation, resulting in reduced carbohydrate levels. Our study found that reducing sugar and total sugar trends across three tobacco phenotypes aligned with prior research. However, starch levels displayed a sustained increase, likely because peak starch accumulation occurs during the technological maturity stage, followed by post-maturity degradation (Cai et al., 2005). Our sampling period preceded technological maturity, explaining the uninterrupted starch rise. Notably, normal mature tobacco leaves accumulated higher starch, reducing sugar, and total sugar contents during development, a trend corroborated by electron microscopy analyses of post-curing leaf tissues. To be precise, sugar can mask undesired odors by producing acids, which can neutralize or attenuate the pungency of the smoke (Yan et al., 2022).The acidic combustion products of elevated reducing sugars in normal mature leaves suppress alkaline compound formation (National Tobacco Quality Supervision and Inspection Center, 2015), thereby maintaining smoke pH balance. These findings underscored the superior carbohydrate accumulation of normal mature tobacco leaves.

Nitrogen metabolism profoundly influences tobacco leaf yield and quality. When tobacco burns, some nitrogen-containing compounds (e.g. proteins, amino acids, etc.) break down into alkaline substances with a high pH. These substances significantly enhance the sensory irritation of the smoke and form a burnt and spicy character, and play a key role in regulating the layering and richness of the aroma of the smoke (Yi et al., 2023). Meanwhile, during tobacco growth and development, excessive nitrogen supply leads to excessive nitrate reductase activity, resulting in nitrogen metabolism and consumption of a large amount of carbohydrates. Moreover, this sugar distribution disorder disrupts the sugar-nitrogen balance, further aggravating chloroplast aging. Chen et al. (2013) reported that nitrogenous compounds decline during leaf senescence, while Zhang et al. (2017) observed gradual alkaloid accumulation (nicotine, nornicotine, anatabine, and anabasine) in developing leaves, with component levels rising with maturity. These trends align with our findings, which further reveal that two lower-quality tobacco leaves accumulated elevated total alkaloids in leaf tip, mid-leaf, and lower leaf zones. Excessive total alkaloids, proteins, and total nitrogen in these phenotypes may impart pungency and irritation to smoke, compromising cigarette palatability and quality.

Beyond its role as a critical fertilizer element promoting economic trait development, nitrogen is essential for chlorophyll biosynthesis and carotenoid accumulation (Wu, 2020). Consistent with this, normal mature tobacco leaves in our study exhibited lower total nitrogen, paralleling their reduced chlorophyll and β-carotene contents. These findings underscore the intricate links between N metabolism, photosynthetic pigment regulation, and alkaloid dynamics in tobacco quality formation.

### 4.3 Mineral balance dynamics

Chlorine (Cl) and potassium (K) are critical mineral elements in tobacco leaves. Xu et al. (2010) demonstrated that imbalanced mineral nutrition (deficiency or excess) disrupts physiological homeostasis and chemical composition harmony in tobacco plants, leading to reduced yield and quality. Tobacco is a K-demanding, Cl-sensitive species. Potassium is the main component of tobacco ash and an important quality factor of tobacco. It can not only improve combustion, flavor and oil content, but also reduce tar content, and plays an important role in improving tobacco utilization rate (Gao et al., 2024). Yang et al. (2022) reported that abnormal Cl levels (either >0.6% or<0.3% in leaves) impair combustibility. At the same time, a mass fraction of 1% or less is appropriate, and a chlorine content above 1% will burn poorly and there will be black ash extinguishing phenomenon, and the quality deteriorates (Tiecher et al., 2023). Our study found that K concentrations consistently exceed Cl levels across all tobacco phenotypes. Notably, multi fertilizer tobacco leaves accumulate elevated Cl (typically >1% across leaf zones) during development. Excessive Cl in lower-quality phenotypes induces leaf thickening, marginal curling, and reduced usability. In contrast, normal mature tobacco leaves maintain moderate K levels, correlating with deep orange coloration, robust aroma, smooth draw, and superior combustibility. The presence of potassium favors the complete combustion of tobacco, while the presence of chlorine has a tendency to slow down combustion, making potash-to-chlorine ratio an important indicator of tobacco combustibility (Yin et al., 2018). This study also found that the potassium-chlorine ratio of multi fertilizer tobacco leaves was the lowest, which adversely affected the quality of tobacco processing in later stage. These findings suggested that Cl/K imbalances in lower-quality tobacco leaves compromised post-maturation quality. Consistently, normal mature tobacco leaves exhibited lower total nitrogen, paralleling their reduced chlorophyll and β-carotene contents, reinforcing the interplay between mineral nutrition, photosynthetic pigment regulation, and sensory quality.

Chemical composition of tobacco leaves serves as a critical indicator of quality status (Peng et al., 2021), with concentration gradients and temporal fluctuations influencing cigarette aroma, sensory quality, and safety (Sun et al., 2011). Even though the natural chemical makeup of tobacco leaves can vary between different batches, sorting and mixing them after harvest helps reduce these differences. However, making sure every batch has the same high quality is still a major challenge for tobacco processors (Yan et al., 2001). Comparative analyses of physiological and biochemical traits among normal mature, multi fertilizer, and reviving tobacco leaves reveal that fertilization regimes significantly modulate maturity and quality-related metrics during tobacco development. Prior research further underscores that tobacco quality is shaped by multifactorial influences, including climatic conditions, geographic provenance, and curing protocols (Dai, 2000). Moreover, biochemical constituents directly dictate processing efficiency and final product quality, explaining why multi fertilizer and reviving phenotypes substantially reduce curing yield and quality. Curing is a pivotal quality determinant, with post-maturation processing parameters exerting profound effects on tobacco outcomes; cultivar-specific curing traits also vary (Sun et al., 2002; Xu et al., 2015). Future studies should prioritize elucidating the physiological mechanisms underlying lower-quality leaf formation and refining cultivation practices (e.g., optimal topping and fertilization) to ameliorate leaf quality. Additionally, cultivar-tailored curing optimizations represent a key strategy for enhancing overall tobacco quality.

## 5 Conclusions

**Figure 8:**
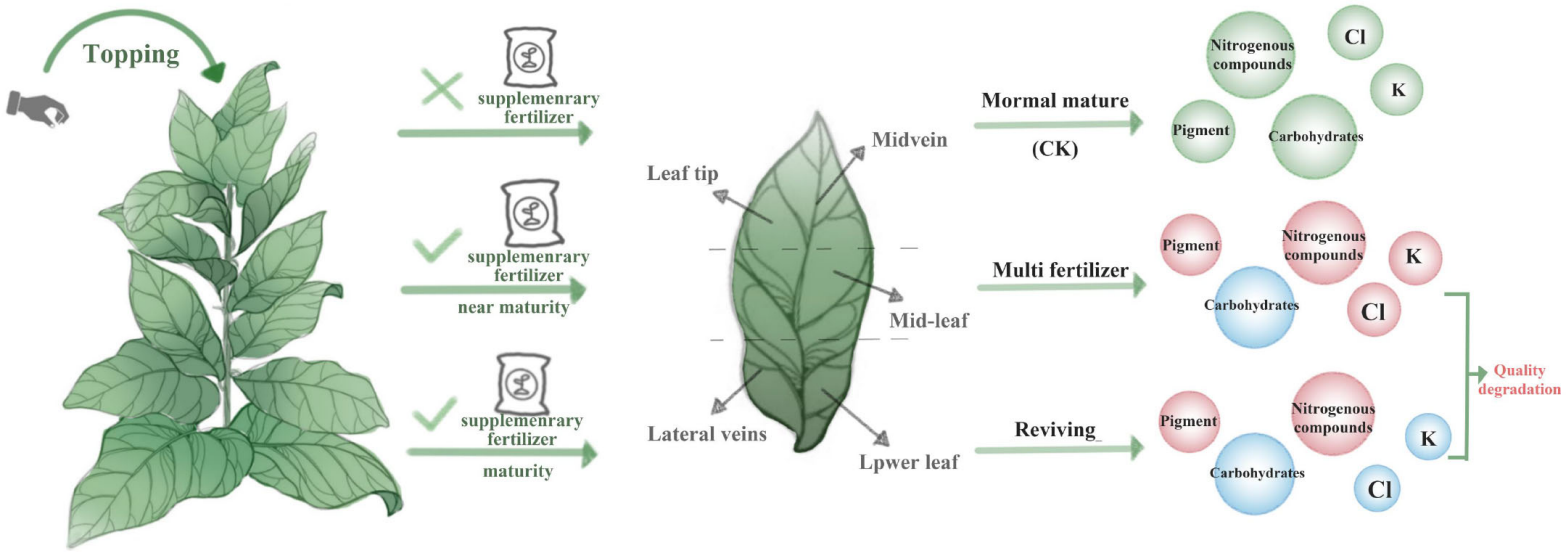
This model figure shows the different quality tobacco leaves produced by different treatments during tobacco development, as well as their physiological and biochemical characteristics in different parts. Red circles indicate the substances that increased compared with the control group, while blue circles indicate the substances that decreased compared with the control group.

During tobacco leaf development, distinct phenotypes exhibit divergent growth traits and biochemical trajectories (See **Figure 8**). Comparative analyses of physiological, colorimetric, pigment, and routine chemical parameters across these phenotypes revealed significant disparities in growth vigor, chromatic evolution, pigment accumulation, and primary metabolite profiles. Relative to normal mature leaves, lower-quality phenotypes displayed elevated pigment retention, resulting in protracted deep green coloration across developmental stages. Normal mature leaves exhibited superior carbohydrate accumulation, with starch, reducing sugars, and total sugars exceeding those of lower-quality phenotypes during all growth stages and the turning point of decline came late. Furthermore, normal mature leaves maintained lower nitrogenous compound levels (total alkaloids, proteins, and total nitrogen), contributing to reduced smoke harshness and enhanced aromatic quality. In terms of mineral nutrition, normal mature leaves accumulated Cl and K in intermediate quantities relative to the extremes observed in lower-quality phenotypes. Two types of low-quality tobacco leaves exhibit lower sugar compound content and higher nitrogenous compound levels across most leaf areas (excluding midvein and lateral veins). These outcomes may be associated with increased N fertilizer application to low-quality tobacco leaves two weeks later. Electron microscopy further revealed cellular structural divergences: multi fertilizer tobacco leaves exhibited fewer and smaller starch granules and loosely arranged thylakoid membranes, delaying leaf maturation. Reviving tobacco leaves displayed early-stage thylakoid lamellae disintegration, followed by reorganization into densely packed arrays during mid-development, though late-stage granal lamellae degradation was observed.

In conclusion, different quality tobacco leaves show different growth characteristics and chemical composition changes in the development process. Normal mature leaves demonstrate superior growth, pigment dynamics, and metabolite regulation, providing a biochemical basis for post-curing quality. Conversely, lower-quality phenotypes require targeted agronomic interventions to mitigate nutrient imbalances and developmental delays. The peak nitrogen uptake period occurs approximately 30 days after tobacco transplanting (during root elongation phase), requiring staged application based on soil mineralization characteristics to prevent excessive concentrated supply. Early growth stages should prioritize nitrate nitrogen to promote vigorous development, while later phases require reduced nitrogen input to prevent leaf retention and delayed maturation. Excessive residual nitrogen in the soil after topping may trigger regrowth, resulting in dense leaf tissue. By regulating nitrogen supply in later growth stages (e.g., maintaining soil moisture at 60% of its water-holding capacity), ineffective nitrogen absorption can be suppressed, facilitating timely yellowing. Future research should investigate cultivation practices (e.g., fertilization timing, topping protocols) to optimize leaf phenotype and quality, thereby informing sustainable tobacco production strategies.

## Data Availability

The original contributions presented in the study are included in the article/supplementary material. Further inquiries can be directed to the corresponding authors.
